# Component 1 Inhibitor Missense (Val480Met) Variant Is Associated With Gene Expression and Sepsis Development in Neonatal Lung Disease

**DOI:** 10.3389/fped.2022.779511

**Published:** 2022-05-20

**Authors:** Enas F. Elngar, Mona A. Azzam, Ayman A. Gobarah, Eman A. Toraih, Manal S. Fawzy, Nouran B. AbdAllah

**Affiliations:** ^1^Department of Pediatrics, Faculty of Medicine, Suez Canal University, Ismailia, Egypt; ^2^Department of Surgery, School of Medicine, Tulane University, New Orleans, LA, United States; ^3^Genetics Unit, Department of Histology and Cell Biology, Faculty of Medicine, Suez Canal University, Ismailia, Egypt; ^4^Department of Medical Biochemistry and Molecular Biology, Faculty of Medicine, Suez Canal University, Ismailia, Egypt; ^5^Department of Biochemistry, Faculty of Medicine, Northern Border University, Arar, Saudi Arabia

**Keywords:** *C1INH*, SNP, gene expression, Real-Time PCR, sepsis, preterm infant

## Abstract

**Background:**

Neonatal lung disease has a multifaceted etiopathology, including an explosive inflammatory sequence in the immature lung. Complement component 1 Esterase INHibitor (C1INH) is implicated in controlling inflammation in response to infection/injury.

**Aim:**

To explore for the first time the association of the *C1INH* rs4926 (Val480Met) variant and circulatory transcript expression levels in the neonates that had evidence of lung disease and the clinic-laboratory data.

**Methods:**

A total of 139 unrelated neonates were enrolled in this case-control study. *C1INH* genotyping and expression analyses were done using TaqMan Genotyping and Real-Time qPCR, respectively.

**Results:**

A/A genotype carriers were two times more likely to develop in newborns with lung disease under homozygote (A/A vs. G/G: OR = 2.66, 95%CI = 1.03-6.87, *p* = 0.039) and recessive (A/A vs. G/G-A/G: OR = 2.42, 95%CI = 1.07-6.06, *p* = 0.047) models. Also, a higher frequency of A/A genotype was observed in the patient's cohort complicated with sepsis (44.2 vs. 14.3%, *p* = 0.002). Neonates with lung disease with A variant had more risk for developing sepsis under homozygote (A/A vs. G/G: OR = 5.19, 95%CI = 1.73-15.6, *p* = 0.002), dominant (A/G-A/A vs. G/G: OR = 2.39, 95%CI = 1.02-5.58, *p* = 0.041), and recessive (A/A vs. G/G-A/G: OR = 5.38, 95%CI = 1.86-15.5, *p* < 0.001) models. Regression analysis revealed rs4926^*^A/A genotype as an independent predictor risk factor for sepsis development in cohorts with lung disease (adjusted OR = 4.26, 95%CI = 1.38-13.1, *p* = 0.012). The circulatory transcript was significantly downregulated in neonates with lung disease in whom rs4926^*^A/A carriers had the least expression levels (median: −2.86, IQR: −3.55 to −1.71; *p* < 0.001). ROC curve analysis revealed *C1INH* expression could differentiate between cohorts with/without subsequent development of sepsis, and the discrimination ability was enhanced when combined with circulatory IL-6 and CRP levels (AUC = 0.926, 95%CI = 0.87-0.97).

**Conclusion:**

The *C1INH* rs4926 variant might play an essential role in the susceptibility to neonatal lung disease and could predict sepsis development in this cohort. Furthermore, the circulatory expression levels of this gene were downregulated in the neonatal lung disease cohort, supporting its potential role in the pathophysiology of this disorder, and highlighting its promising role in future targeted therapy.

## Introduction

There are multiple causes of neonatal lung disease. The most common is neonatal respiratory distress, which is pulmonary insufficiency that commences at or shortly after birth and increases over the first 2 days of life. Clinically, neonates with respiratory distress present with early respiratory distress comprising cyanosis, grunting, retractions, and tachypnea and may result in respiratory failure (1, 2). Neonatal respiratory distress is a severe disorder that most afflict premature infants. It accounts for 10% of neonatal deaths, making it the fifth leading cause of death in the first year of life even with interventions (3).

Neonatal respiratory distress is caused by structural and functional immaturity of the lung with a deficiency of producing enough alveolar surfactant, resulting in decreased lung compliance and unstable alveoli (4). Pathologically, the disease is characterized by epithelial cell injury, edema formation, and intra-alveolar contact phase activation (also called plasma kallikrein-kinin system) (5, 6).

Although premature infants have benefited from recent advances in surfactant therapy, among the survivors treated with currently available surfactants, a significant percentage of survived infants grow up with a recurrent pulmonary infection and chronic inflammation. Given that preterm infants are particularly poor at mounting an antibody response, innate immunity mechanisms in this patient group may be especially important. Thus, there was a need to develop alternative therapeutic strategies (4). A combination of *C*omplement component *1* Esterase *INH*ibitor (C1INH) and lung surfactant or immunoglobulins for the treatment of respiratory disorders as Infant Respiratory Distress Syndrome (IRDS), Adult Respiratory Distress Syndrome (ARDS), and acute lung injury (ALI) was tested under several trials (7–9).

C1INH (https://www.uniprot.org/uniprot/P05155), also known as Serpin Peptidase Inhibitor Clade G Member 1 (SERPING1), has gained increasing attention as a regulator of several systems such as the complement system, plasma kallikrein of the contact system (which plays a central role in RDS etiopathology), the coagulation system (factor XIIa, XIa, and thrombin), and the fibrinolytic system through the inhibition of plasmin, and tissue-type plasminogen activator (10).

It is encoded by the *C1INH* gene located at the long arm of chromosome 11 (11q12.1), and its nine exons encode the most highly glycosylated plasma protein in the circulation, with 35% of its weight constituted by carbohydrates (11). It is synthesized primarily in the liver as a single amino acid chain, and its deficiency is associated with hereditary angioneurotic edema (HANE) type I or II. C1INH is a serine protease inhibitor (serpin). Serpins help control several types of chemical reactions by blocking the activity of specific proteins (12). It plays an emanant role in controlling a range of processes involved in maintaining blood vessels, platelet degranulation, regulating inflammation in response to infection or injury, blood coagulation, fibrinolysis, and the generation of kinins (13).

Subcutaneous injection of a plasma-derived concentrate of C1INH was used in adult and pediatric patients with HANE to compensate for low levels or improper function of C1INH protein. Indeed, C1INH treatment has improved the outcome in several disease models, including sepsis and bacterial infection, ischemia-reperfusion injury, hyper-acute transplant rejection, and other inflammatory disease models (14, 15). Malgorzata et al. studied its ability to control the inflammatory processes *in vitro* and *in vivo* to decipher the possible underlying C1INH-related mechanisms mediating protection. They found that C1INH administration could provide a new therapeutic option for disorders associated with histone release (5). Collectively, these findings support the potential implication of C1INH in respiratory-related distress and open a new era for exploring its utility in the clinic.

The current authors in the clinical work observed variations in neonatal lung disease incidence and clinical presentation/response to postnatal surfactant therapy and optimal ventilator care in both full-term and preterm neonates; hence a speculated role of genetic element, in addition to the environmental triggers, was raised. Given the well-known impact of genetic variants, including the single nucleotide polymorphisms (SNPs), on gene expression and activity of the encoded proteins which could influence disease susceptibility and phenotype (16, 17), the authors were inspired to explore for the first time the possible association of *C1INH* rs4926 gene variant and expression with neonatal lung disease risk and the clinical features in an Egyptian neonate cohort. The results of this work could help in risk stratification of this vulnerable population with early implementation of the appropriate preventive/therapeutic strategies.

## Subjects and Methods

### Study Population

A total of 139 consecutive unrelated newborns were recruited from the neonatal intensive care unit (NICU) of Suez Canal University Hospitals, Ismailia, Egypt, which is a level III NICU. All neonates with lung disease were included. All preterm admitted from June 2018 to July 2019 were screened to be enrolled in the study based on the guidelines (1). Premature newborns with comorbidities or congenital malformations were excluded. The patient group (*n* = 94) included preterm newborns (28 < 37 weeks GA) who developed respiratory distress (based on clinical manifestations/chest X-ray findings) in the first 6 h of life. It is worth noting that the more mature neonates did have lung disease, but a much milder form related to them being near-term or infants of diabetic mothers. None of them required invasive or non-invasive ventilation, as seen in Table 1.

**Table 1:** Characteristics of the study population.

**Characteristics**	**Levels**	**Controls**	**Neonates with lung disease**	***P*-value**
		**(*n* = 45)**	**(*n* = 94)**	
**Demographics**				
Sex	Female	16 (35.6)	42 (44.7)	0.36
	Male	29 (64.4)	52 (55.3)	
Consanguinity	Negative	39 (86.7)	84 (89.4)	0.77
	Positive	6 (13.3)	10 (10.6)	
Order of baby	1^st^	10 (22.2)	30 (31.9)	0.42
	2^nd^	14 (31.1)	32 (34.0)	
	3^rd^	17 (37.8)	25 (26.6)	
	4^th^ or more	4 (8.8)	7 (7.4)	
**Antepartum follow-up**				
PROM	Positive	0 (0.0)	25 (26.6)	**<0.001**
Antepartum hemorrhage	Positive	0 (0.0)	4 (4.3)	0.30
Maternal diabetes	Positive	1 (2.2)	3 (3.2)	0.74
Maternal SLE	Positive	0 (0.0)	1 (1.1)	0.48
Bronchial asthma	Positive	1 (2.2)	0 (0.0)	0.32
Maternal anemia	Positive	0 (0.0)	2 (2.1)	0.32
Maternal hypertension	Positive	1 (2.2)	5 (5.3)	0.66
Pre-eclampsia	Positive	0 (0.0)	10 (10.6)	**0.030**
Eclampsia	Positive	0 (0.0)	2 (2.1)	0.32
Triple I	Positive	0 (0.0)	8 (8.5)	**0.044**
Preterm labor pain	Positive	10 (22.2)	19 (20.2)	0.84
**Delivery**				
Mode of delivery	Vaginal	22 (48.9)	40 (42.6)	0.58
	Cesarean	23 (51.1)	54 (57.4)	
Preterm delivery	Negative (≥37 weeks)	35 (77.8)	29 (30.9)	**<0.001**
	Preterm (32-36 weeks)	10 (22.2)	48 (51.1)	
	Extreme preterm (28-31 weeks)	0 (0.0)	17 (18.1)	
GA at birth, weeks	Mean ± SD	37.6 ± 1.21	33.9 ± 2.24	**<0.001**
Birth weight, Kg	Mean ± SD	3.03 ± 0.5	1.98 ± 0.5	0.16
Multiple gestations	Single	45 (100)	84 (89.4)	**0.030**
	Multiple	0 (0.0)	10 (10.6)	
**Inflammatory markers**				
Interleukin-6 (log2FC)	Median (IQR)	0.0	2.6 (2.1-3.26)	**<0.001**
C-reactive protein (mg/L)	Median (IQR)	0.4 (0.3-0.6)	19.0 (3.0-60)	**<0.001**

*Data are presented as number and percentage, mean and standard deviation (SD), or median and interquartile range (IQR). Two-sided Chi-square, Mann-Whitney U, or Student's t-tests were used. PROM, premature rupture of membrane; SLE, systemic lupus erythematosus; GA, gestational age. Bold values indicate significance at p < 0.05*.

The severity of respiratory distress was assessed clinically by Downes' scoring system (score < 5 was regarded as mild, 5-7 as moderate, and ≥8 was regarded as severe respiratory distress) and radiologically according to chest X-ray findings (stage 1-4) (18). Of the neonates with lung disease, 52 developed late-onset sepsis (onset > 72 h). This was diagnosed based on clinical and laboratory findings, including apnea, cyanosis, increased respiratory demand, lethargy, hypotonia, feeding intolerance, abdominal distension, reduced variability and transient decelerations in heart rate, temperature instability, seizures, and bulging fontanels. Laboratory findings included a combination of multiple biomarkers, such as the total number of neutrophils, immature to total neutrophil ratio, and C-reactive protein (CRP) and blood culture (19). None of the included newborns suffered from acute respiratory distress syndrome following sepsis or pneumonia. The control group (*n* = 45) included preterm and full-term neonates without lung disease or other comorbidities, recruited from the same hospital as the cases. We included full-term neonates in the control group due to the paucity of healthy premature neonates and the difficulty of taking consent from parents to include their premature neonates as controls.

### Clinical Assessment and Data Collection

A comprehensive questionnaire including variables in neonatal lung disease development was designed and distributed among the neonatologists in the NICU. Maternal and neonatal histories were evaluated for the following clinical data: birth weight, gestational age, gender, order of birth, mode of delivery, antenatal glucocorticoid treatment, the reason for preterm birth, and severity of the respiratory distress, and any chronic maternal illness, maternal premature rupture of membrane (PROM), and history of medications.

Informed written consent was obtained from parents. The diagnosis of respiratory distress was made by the attending physician (neonatologist) based on classic clinical presentations and radiographic findings (20). Premature infants were classified by weight into low birth weight (LBW) <2,500 g, very low birth weight (VLBW) <1,500 g, extremely low birth weight (ELBW) <1,000 g (only four neonates <28 weeks GA were admitted due to emergency delivery and difficult transportation). All the newborns with respiratory distress underwent therapeutic and supportive care, surfactant administration, and mechanical ventilation as per hospital protocol, following the European consensus guidelines (21).

### Sample Collection and Laboratory Analysis

Venous blood samples were collected on several occasions under aseptic conditions into vacutainer plain/EDTA tubes. The required samples (5 ml) were collected at once only in case of UVC inserted and the blood drained for metabolic studies or other investigations, under the supervision of the neonatologist in charge). The plain tubes were sent for routine chemical analysis in the chemistry lab of the specified hospital. The EDTA tubes were used for subsequent genomic analysis. About 2 ml blood was inoculated directly into blood culture medium specific vials with double-checking patient identification label and sent to the microbiology laboratory for cultivation. Results of subcultures examined for growth after incubation of 48 h were delivered as preliminary results; blood culture was considered sterile after 7 days of negative cultivation results (22).

### Chest X-Ray Assessment

Grading respiratory distress was done as follows: Grade I: Slightly reticular (granular) pattern, decrease the transparency of lung, Grade II: soft decrease in transparency with air bronchogram, Grade III: Like grade II with a more substantial decrease in transparency with blurry diaphragm and heart, Grade IV: White lung, with homogenous lung opacity (20).

### Molecular Analysis

#### Bioinformatic Selection of *C1INH* Gene Variant

According to the PubMed, ClinVar, and VarSome databases, 244 genetic variants with known pathogenicity were reported, including 172 (70.5%) pathogenic, 40 (16.4%) uncertain significance, and 32 (13.1%) benign variants (Supplementary Figure S1D). The pathogenic variant with the highest allele frequency is rs4926 (G/A) at chr11:57614516, a missense mutation with minor allele frequency (MAF) of 0.15 caused by the substitution of valine (GTG) with methionine (ATG) at codon 480. Both residues are medium size and hydrophobic and have similar physicochemical properties. This polymorphism overlaps 20 transcripts and is predicted to have benign consequences (PolyPhen 0.034) except for ENST00000403558.1 transcript (PolyPhen 0.557 with possibly damaging effect).

#### Nucleic Acids Extraction

Nucleic acids were isolated from whole blood using the ABIOpure Total DNA and RNA isolation kit (AllianceBio, Catalog no. M501DP100 and M541RP50-B, respectively) following the manufacturer-supplied protocol. Nucleic acid concentration and purity at the absorbance ratio 260/280 nm were determined by NanoDrop ND-1000 spectrophotometer (NanoDrop Tech., Inc. Wilmington, DE, USA).

#### Discrimination Analysis of *C1INH* rs4926 Variant

DNA samples of patients and controls were genotyped for *C1INH* rs4926 polymorphism using Real-Time polymerase chain reaction allelic discrimination technology and TaqMan assay (Applied Biosystems, assay ID C__7498220_10) with FAM/VIC dyes (G/A: V480M) (Supplementary Figure S1) as detailed previously (23, 24).

### *C1INH* Gene Expression Analysis

The High-Capacity cDNA Reverse Transcription (RT) Kit (Applied Biosystems, P/N 4368814) was used to convert RNA into cDNA. RT was carried out in T-Professional Basic, Biometra PCR System (Biometra, Goettingen, Germany). *C1INH* expression was quantified using TaqMan® assay specific for *C1INH* gene (Applied Biosystems, assay ID Hs001613781_m1) and Taqman® Universal PCR master mix (Applied Biosystems, P/N 4440043) compared to glyceraldehyde 3-phosphate dehydrogenase (*GAPDH*) expression (Assay ID Hs402869) as an endogenous control. The PCR program was run as described previously (25). The selected assay covered 17 splice variants of the *C1INH* gene, including ENST00000378324.6 transcript enriched in the circulation. Appropriate controls were run in each duplicated experiment. Fold change was calculated using the delta-delta threshold cycle equation (26). We followed all the necessary quality control measures recommended by the “Minimum Information for Publication of Quantitative Real-Time PCR Experiments; MIQE” guidelines (27).

### Statistical Analysis

Data were managed using the R software version 4.0.4/RStudio 1.4.1106, GraphPad prism v.8.0, STATA v.16, and “Statistical Package for the Social Sciences (SPSS) for Windows” version 27.0. Categorical variables were compared using the chi-square (χ^2^) or Fisher's exact tests. Data are represented as numbers and percentages. At the same time, student's *t*-test, Mann-Whitney *U* (MW), and Kruskal-Wallis (KW) tests were used to compare continuous variables according to data distribution and variance homogeneity that was checked by “Shapiro-Wilk test” and “Levane test,” respectively, to compare continuous variables. Data were expressed as mean ± standard deviation (SD) or median and interquartile range (IQR).

The allele frequency within each group was determined as the number of occurrences of an individual allele divided by the total number of alleles. Minor allele frequency (MAF) was compared to other ethnic populations in the 1000 Genome Project (www.ensembl.org). Genotype frequency (G/G, A/G, and A/A) per each group was estimated (28). Hardy-Weinberg equilibrium (HWE) for the SNP was tested by using a goodness-of-fit χ2-test *via* the Online Encyclopedia for Genetic Epidemiology (OEGE) software (http://www.oege.org/software/hwe-mr-calc.shtml). The associations of rs4926 genotype and susceptibility to neonatal lung disease were computed for various genetic association models, including heterozygote comparison (A/G vs. G/G), homozygote comparison (A/A/ vs. G/G), dominant model (A/G-A/A vs. G/G), and recessive model (A/A vs. A/G-G/G). Adjusted odds ratio by sex and gestational age was reported along with their 95% confidence intervals (CI). A two-tailed *P-*value < 0.05 was considered statistically significant.

Fold change of *C1INH* gene expression was calculated using quantitative threshold level of patients and controls following the LIVAK method (26). Logarithmic transformation was used in plotting the data. The receiver operating characteristic (ROC) curves were applied to get the best cutoff values of the *C1INH* gene level for discriminating patients from controls. The area under the Curve (AUC), sensitivity, and specificity was identified. Iteration in combination with Interleukin-6 (IL-6) and C reactive protein (CRP) levels was performed.

A binary logistic regression test was performed to identify independent risk factors for neonatal sepsis after adjusting confounding parameters. Significant variables in the univariate analysis were selected. Other variables reported having a role in prior literature were added. Those with strong correlations were excluded, and only one was selected. Cox Proportionate Hazards regression analysis was performed to identify the predictors for neonatal mortality. Data were reported as hazard ratio and 95% confidence interval (CI).

## Results

### Characteristics of the Study Population

Total 94 neonates were admitted to the Neonatal Intensive Care Unit (NICU) for respiratory distress during the study period. The study included 94 neonatal lung disease cases (42 females and 52 males) and 45 controls (16 females and 29 males). Preterm delivery was reported in 65 neonates with lung disease (69.1%). Premature rupture of membrane (PROM) was the most common predisposing factor associated with the development of neonatal lung disease in 25.6% of newborns, followed by preterm labor pain (20.2%). Significant elevation of inflammatory markers was remarkable in serum of neonatal lung disease patients (*p* < 0.001) (Table 1). The overall mortality rate due to respiratory distress was 39.4 (*n* = 37).

### Characteristics of Neonatal Lung Disease With and Without Sepsis

Of the neonates with lung disease, 52 (55.3%) developed sepsis, 51.9% were females. Blood culture revealed *Klebsiella* (11.7%), *Staphylococcus aureus* (7.4%), *Streptococcus* (1.1%), and *Pseudomonas* (3.2%) infection, for which specific neonatal antibiotics were given. Neonates with lung disease with subsequent sepsis were more likely to be delivered preterm (84.6 vs. 50%, *p* = 0.001) and to have less birth weight (78.8 vs. 59.6%, *p* < 0.001). Septic neonate had significantly lower levels of hemoglobin (median = 13, IQR = 11.6-15 in sepsis group vs. median = 14.9, IQR = 12-16.6 in non-sepsis group, *p* = 0.005) and serum albumin (median = 2.6, IQR = 2.3-3.0 vs. median = 3.2, IQR = 2.8-3.5, respectively, *p* = 0.002). In contrast, sepsis cohorts had significantly higher levels of C-reactive protein [52.8 (34-73.3) vs. 2.60 (1-3.65), *p* < 0.001] (Table 2).

**Table 2:** Characteristics of neonates with lung disease cohort according to subsequent complications with sepsis.

**Characteristics**	**Levels**	**Total** **(n = 94)**	**No sepsis** **(n = 42)**	**Sepsis** **(n = 52)**	***p-*value**
**Demographics**					
Sex	Female	42 (44.7)	15 (35.7)	27 (51.9)	0.14
	Male	52 (55.3)	27 (64.3)	25 (48.1)	
Consanguinity	Negative	84 (89.4)	40 (95.2)	44 (84.6)	0.17
	Positive	10 (10.6)	2 (4.8)	8 (15.4)	
**Presentation**					
Mode of delivery	Vaginal	40 (42.6)	15 (35.7)	25 (48.1)	0.29
	Cesarean	54 (57.4)	27 (64.3)	27 (51.9)	
Preterm delivery	Negative (≥37 weeks)	29 (30.9)	21 (50.0)	8 (15.4)	**0.001**
	Preterm (32-36 weeks)	48 (51.1)	13 (31.0)	35 (67.3)	
	Extreme preterm (28-31 weeks)	17 (18.1)	8 (19.0)	9 (17.3)	
Cause of preterm delivery	PROM	25 (26.6)	10 (23.8)	15 (28.8)	0.64
	Pre-eclampsia	12 (12.7)	5 (11.9)	7 (13.4)	0.42
	Triplet I	8 (8.5)	4 (9.5)	4 (7.7)	0.75
	Antepartum hemorrhage	4 (4.3)	2 (4.8)	2 (3.8)	0.82
	Preterm labor pain	19 (29.2)	17 (40.5)	2 (8.7)	**0.012**
Degree of BW	LBW (2.499-2.000 gm)	38 (40.4)	27 (64.3)	11 (21.2)	**<0.001**
	LBW (2.000-1.500 gm)	40 (42.5)	7 (16.7)	33 (63.5)	
	VLBW (<1.500-1.000 gm)	12 (12.7)	6 (14.3)	6 (11.5)	
	ELBW (<1.000 gm)	4 (4.2)	2 (4.8)	2 (3.8)	
Multiple gestations	Single	84 (89.4)	40 (95.2)	44 (84.6)	0.17
	Multiple	10 (10.6)	2 (4.8)	8 (15.4)	
Maternal comorbidities	Maternal diabetes	3 (3.2)	0 (0.0)	3 (5.8)	0.25
	Maternal anemia	2 (2.1)	2 (4.8)	0 (0.0)	0.19
	Maternal hypertension	5 (5.3)	2 (4.8)	3 (5.8)	0.82
**Investigation**					
Complete blood picture	WBCs X 10^3^	7.5 (5.1-12.8)	7.6 (5.36-11.9)	7.25 (5-13.7)	0.92
	Platelets X10^3^	195 (127-303)	202 (147-288)	189 (116-310)	0.88
	Hemoglobin g/dl	13.8 (12-15.8)	14.9 (12-16.6)	13 (11.6-15)	**0.005**
Blood chemistry	Na mEq/L	140 (137-145)	142 (139-145)	139 (134-145)	0.06
	K mmol/L	4.7 (4.30-5.1)	4.9 (4.4-5)	4.5 (4.1-5.1)	0.43
	Ca mg/dl	8.7 (89-9.2)	8.7 (8.10-9.2)	8.7 (8-9.1)	0.90
	Creatinine mg/dl	0.5 (0.3-0.61)	0.5 (0.30-0.60)	0.5 (0-0.3)	0.54
	PH	7.2 (7.2-7.3)	7.1 (7.17-7.2)	7 (7.25-7.3)	0.22
	PO_2mmHg_	70 (59-80)	58 (54-67)	72.5 (59-80)	0.40
	PCO_2mmHg_	47 (28.6-55)	39 (27-40)	48 (29.6-55)	0.42
	HCO_3mEq/L_	180 (16-20)	17.5 (17.3-18)	18.2 (16-20)	0.47
Liver function test	ALT, U/L	120 (8-16)	12 (8-17)	13.5 (9-16)	0.74
	AST, U/L	35 (30-60)	35 (28-58)	40.5 (30-64)	0.31
	Albumin, g/dl	3.0 (2.7-3.4)	3.20 (2.80-3.5)	2.6 (2.3-3)	**0.002**
Inflammatory markers	CRP mg/L	19 (3-60)	2.60 (1-3.65)	52.8 (34-73.3)	**<0.001**
	IL6	2.6 (2.1-3.26)	2.7 (2.05-3.4)	2.6 (2.1-3.04)	0.47
Chest X ray	No evidence	8 (8.5)	4 (9.5)	4 (7.7)	0.21
	Grade 1	57 (60.6)	22 (52.4)	35 (67.3)	
	Grade 2,3	18 (19.1)	8 (19.0)	10 (19.2)	
	Grade 4	11 (11.7)	8 (19.0)	3 (5.8)	
**Management**					
Maternal medications	Antenatal antibiotics	6 (6.4)	1 (2.4)	5 (9.6)	0.22
	Antenatal steroids	23 (24.5)	6 (14.3)	17 (32.7)	**0.039**
	Single dose steroids	9 (39.1)	3 (7.1)	6 (11.5)	0.15
	Two doses' steroids	6 (26.1)	2 (4.8)	4 (7.7)	
	Three doses' steroids	8 (34.8)	1 (2.4)	7 (13.5)	
Ventilation	O_2_ therapy	58 (61.7)	24 (57.1)	34 (65.3)	0.18
	Non-invasive MV (CPAP)	23 (24.5)	12 (28.6)	11 (21.2)	
	Invasive MV (SIMV)	13 (13.8)	6 (14.3)	7 (13.5)	
Surfactant	Positive	9 (9.6)	6 (14.3)	3 (5.8)	0.29
**Outcomes**					
Duration, days	Ventilation	4 (2-7)	3 (1-4)	7 (4-9)	**0.007**
	NICU	10 (7-14.8)	7 (5-12)	12 (9.7-17)	**0.001**
	Total length of stay	10.5 (7-15)	7 (5-12)	13 (10-13)	**0.001**
Survival	Died	37 (39.4)	18 (42.9)	19 (36.5)	0.67

*PROM, premature rupture of membrane; GA, gestational age; BW, birth weight; VAP, ventilation-association pneumonia; LBW, low birth weight; VLBW, very low birth weight; ELBW, extremely low birth weight; WBC, white blood cells; ALT, alanine transaminase; AST, aspartate transaminase; CRP, C-reactive protein; IL6, interleukin 6; MV, mandatory ventilation; CPAP, continuous positive airway pressure; SIMV, synchronized intermittent mandatory ventilation; NICU, neonatal intensive care unit. Two-sided Chi-square, Mann-Whitney U, or Student's t-tests were used to compare sepsis and non-sepsis groups. Bold values indicate significance at p < 0.05*.

Neonates with sepsis required more prolonged mechanical ventilation duration [7 days (4–9) vs. 3 days (1–4), *p* = 0.007], and was associated with longer NICU [12 days (9.7-17) vs. 7 days (5–12), *p* = 0.001] and total hospital stays [13 days (10–13) vs. 7 days (5–12), *p* = 0.001]. However, there was no significant difference in their mortality rate (36.5% in sepsis vs. 42.9% in the non-sepsis group, *p* = 0.67 (Table 2).

Complications rather than sepsis were also recorded in our cohort, affecting 65 (69.1%) neonates. Air leak syndromes presented in 10 (15.4%) neonates, pulmonary hemorrhage affected five preterms, and all passed away (7.7%), and eight babies had necrotizing enterocolitis (grades I and II; 12.3%). Infiltration extravasation injury caused by vancomycin and TPN (total parenteral nutrition) has disturbed four (6.15%) neonates, and only one needed plastic surgery treatment. About five (9.23%) premature neonates suffered PPHN (persistent pulmonary hypertension), for which they received medical treatment and responded. Periventricular hemorrhage was detected in seven (10.7%) cases (germinal matrix hemorrhage grade I and grade II by cranial ultrasound), and retinopathy of prematurity (ROP) grades I and II were diagnosed in five (7.7%) neonates.

### *C1INH* Gene Variant Analysis

**Figure 1:**
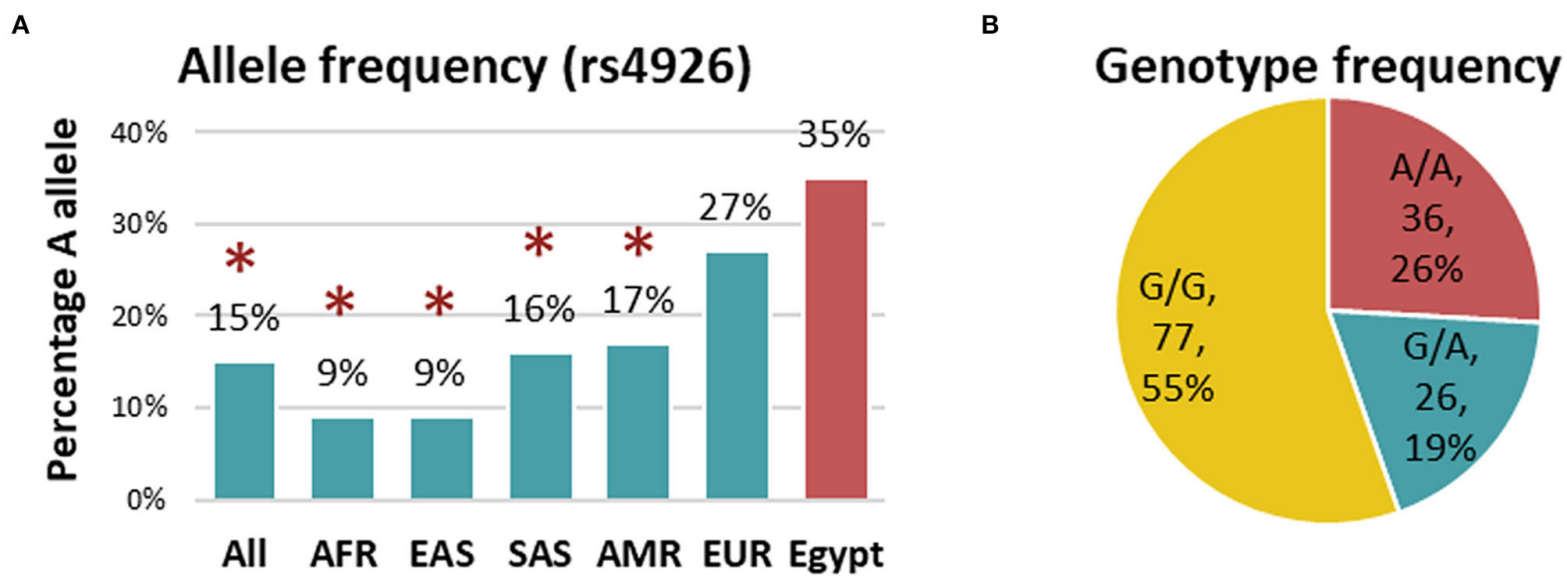
Genotype and allele frequency of *C1INH* rs4926 polymorphism in the studied population. **(A)** Allele frequency of rs4926 G/A polymorphism in the current study (red) compared to other populations (green) reported in 1000 Genome Project. AFR, African; EAS, East Asian; SAS, South Asian; AMR, American; EUR, European. A two-sided Chi-square test was used. [Data source: ensembl.org]. **(B)** Genotype frequency of rs4926 (c.1438G > A) across the whole cohorts. *Indicates the p-value < 0.05.

Genotyping of rs4926 polymorphism in 139 subjects revealed a minor allele frequency (A allele) of 0.35 among the whole cohorts. This frequency was significantly higher than Africans, Asians, and Americans reported in the 1000 Genome Project (Figure 1A). Similarly, G/G was the most frequent genotype in the study population accounting for 55% (Figure 1B). Genotype frequencies followed Hardy-Weinberg equilibrium (*p* = 0.76 for controls and 0.60 for cases). As depicted in Table 3, the A/A genotype was more frequent in neonates with lung disease than controls (30.9 vs. 15.6%, *p* = 0.039). Homozygote carriers for the A allele were two times more likely to develop neonatal lung disease under homozygote comparison (A/A vs. G/G: OR = 2.66, 95%CI = 1.03-6.87, *p* = 0.039) and recessive model (A/A vs. G/G-A/G: OR = 2.42, 95%CI = 1.07-6.06, *p* = 0.047).

**Table 3:** Genetic association models for neonatal respiratory distress risk assessment.

**Model**	**Genotype**	**Controls**	**Neonates with respiratory distress**	**Adjusted OR (95%CI)**	***P*-value**
		**(*n* = 45)**	**(*n* = 94)**		
Codominant	G/G	30 (66.7%)	47 (50%)	Reference	
	A/G	8 (17.8%)	18 (19.1%)	1.52 (0.58-3.97)	0.45
	A/A	7 (15.6%)	29 (30.9%)	**2.66 (1.03**-**6.87)**	**0.039**
Dominant	G/G	30 (66.7%)	47 (50%)	Reference	
	A/G-A/A	15 (33.3%)	47 (50%)	2.06 (0.98-4.34)	0.06
Recessive	G/G-A/G	38 (84.4%)	65 (69.2%)	Reference	
	A/A	7 (15.6%)	29 (30.9%)	**2.42 (1.07**-**6.06)**	**0.047**
Over-dominant	G/G-A/A	37 (82.2%)	76 (80.8%)	Reference	
	A/G	8 (17.8%)	18 (19.1%)	1.15 (0.46-2.93)	0.85

*Data are presented as numbers and percentages. A two-sided Chi-square test was employed. Adjusted odds ratio (OR) and 95% confidence interval (CI) by sex and gestational age were estimated for different inheritance models using logistic regression analysis. Bold values indicate significance at p < 0.05*.

### Association of *C1INH* Polymorphism With Clinical and Laboratory Features

In the neonatal lung disease group, G/G, A/G, A/A genotypes were found in 47 (50%), 18 (19.1%), and 29 (30.9%) patients, respectively. As illustrated in Table 4, despite preterm neonates with homozygote mutant allele (A/A) for methionine residue having the highest gestational age (*p* = 0.049) and birth weight (*p* = 0.006), they exhibited the least hemoglobin level (*p* = 0.002) and higher frequency of sepsis (*p* = 0.008). There was no significant difference in other clinical and laboratory parameters. In addition, there was no differential difference in patients' outcomes, including the timing of weaning from mechanical ventilation (*p* = 0.58), the mortality rate (*p* = 0.26), or hospital stay (*p* = 0.12).

**Table 4:** Association of *C1INH* polymorphism with clinical and laboratory characteristics of neonatal lung disease cohort.

**Characteristics**	**Levels**	**G/G (*n* = 47)**	**A/G (*n* = 18)**	**A/A (*n* = 29)**	***P*-value**
**Demographics**					
Sex	Female	22 (46.8)	7 (38.9)	13 (44.8)	0.84
	Male	25 (53.2)	11 (61.1)	16 (55.2)	
Consanguinity	Positive	5 (10.6)	0 (0.0)	5 (17.2)	0.17
**Presentation**					
Mode of delivery	Vaginal	19 (40.4)	8 (44.4)	13 (44.8)	0.91
	Cesarean	28 (59.6)	10 (55.6)	16 (55.2)	
Gestational age, weeks	Median (IQR)	34 (32-36)	35 (33-36)	35 (34-36)	**0.049**
Birth weight, Kg	Median (IQR)	1.68 (1.5-2.06)	1.96 (1.68-2.5)	2.2 (1.76-2.6)	**0.006**
Preterm labor pain	Positive	9 (36.0)	1 (7.7)	9 (33.3)	0.15
PROM	Positive	11 (23.4)	6 (33.3)	8 (27.6)	0.71
Preeclampsia/fits	Positive	4 (8.5)	5 (27.7)	3 (10.3)	0.10
Triple I	Positive	5 (10.6)	1 (5.6)	2 (6.9)	0.75
**Investigation**					
Laboratory findings	Positive blood culture	13 (27.7)	5 (27.8)	4 (13.8)	0.54
	WBCs, x10^3^	7.5 (5.3-12.1)	7.1 (4.6-13.5)	8.3 (5.0-13.3)	0.83
	Platelets, x10^3^	184 (104-264)	211 (161-321)	271 (134-328)	0.09
	Hemoglobin, g/dL	14.9 (12.4-16.3)	12.9 (12.3-14.9)	12.4 (9.2-14.7)	**0.002**
	CRP, mg/L	4.6 (2.0-60.1)	7.0 (2.7-60.0)	39.6 (16-61.1)	0.21
	IL6-fold change	2.54 (1.7-3.2)	3.08 (2.1-3.5)	2.69 (2.3-3.1)	0.42
Chest X ray	Grade 2	6 (12.8)	6 (33.3)	6 (20.7)	0.33
	Grade 3	4 (8.5)	2 (11.1)	5 (17.2)	0.35
**Management**					
Maternal medications	Antepartum steroids	11 (23.4)	6 (33.3)	6 (20.7)	0.60
Ventilation	Non-invasive MV (CPAP)	11 (23.4)	4 (22.2)	8 (27.6)	0.84
	Invasive MV (SIMV)	6 (12.8)	3 (16.7)	4 (13.8)	0.79
Surfactant	Positive	6 (12.8)	1 (5.6)	2 (6.9)	0.56
**Outcomes**					
Duration, days	Ventilation	4.5 (2.0-7.0)	3.0 (1.0-6.0)	4.0 (2.0-7.0)	0.58
	NICU	9.0 (5.0-12.0)	10 (7.0-17.5)	13 (9.0-17.0)	0.13
	Total length of stay	9.0 (5.0-13.0)	10 (7.0-17.0)	13 (9.0-17.0)	0.12
	Time to death	9.0 (4.5-14.5)	9.5 (4.5-17.5)	12.5 (7.0-17.3)	0.64
Sepsis	Negative	26 (55.3)	10 (55.6)	6 (20.7)	**0.008**
	Positive	21 (44.7)	8 (44.4)	23 (79.3)	
Survival	Survived	31 (66.0)	12 (66.7)	14 (48.3)	0.26
	Died	16 (34.0)	6 (33.3)	15 (51.7)	

*Data are presented as the median and interquartile range (IQR). PROM, premature rupture of membrane; WBC, white blood cells; CRP, C-reactive protein; IL6, interleukin 6; MV, mandatory ventilation; CPAP, continuous positive airway pressure; SIMV, synchronized intermittent mandatory ventilation; NICU, neonatal intensive care unit. Two-sided Chi-Square and Kruskal-Wallis tests were used. Bold values indicate significance at p < 0.05*.

### Methionine Mutation Increased the Risk of Sepsis in Neonatal Lung Disease Patients

Within the neonatal lung disease group, 55.5% (*N* = 52) developed sepsis. A higher frequency of A/A genotype was observed in premature neonates complicated with sepsis (44.2 vs. 14.3%, *p* = 0.002). Neonates with lung disease with A variant had more risk for developing sepsis than their counterparts under homozygote comparison (A/A vs. G/G: OR = 5.19, 95%CI = 1.73-15.6, *p* = 0.002), dominant model (A/G-A/A vs. G/G: OR = 2.39, 95%CI = 1.02-5.58, *p* = 0.041), and recessive model (A/A vs. G/G-A/G: OR = 5.38, 95%CI = 1.86-15.5, *p* < 0.001) (Table 5).

**Table 5:** Genetic association models for risk of developing sepsis in neonatal lung disease cohort.

**Model**	**Genotype**	**No sepsis (*n* = 42)**	**Sepsis (*n* = 52)**	**Adjusted OR (95%CI)**	***P*-value**
Codominant	G/G	26 (61.9%)	21 (40.4%)	Reference	
	A/G	10 (23.8%)	8 (15.4%)	0.87 (0.28-2.69)	0.98
	A/A	6 (14.3%)	23 (44.2%)	**5.19 (1.73-15.61)**	**0.002**
Dominant	G/G	26 (61.9%)	21 (40.4%)	Reference	
	A/G-A/A	16 (38.1%)	31 (59.6%)	**2.39 (1.02-5.58)**	**0.041**
Recessive	G/G-A/G	36 (85.7%)	29 (55.8%)	Reference	
	A/A	6 (14.3%)	23 (44.2%)	**5.38 (1.86-15.55)**	**<0.001**
Over-dominant	G/G-A/A	32 (76.2%)	44 (84.6%)	Reference	
	A/G	10 (23.8%)	8 (15.4%)	0.51 (0.17-1.47)	0.21

*Data are presented as numbers and percentages. A two-sided Chi-square test was employed. Adjusted odds ratio (OR) and 95% confidence interval (CI) by sex and gestational age were estimated for different inheritance models using logistic regression analysis. Bold values indicate significance at p < 0.05*.

### *C1INH* Gene Expression Analysis

**Figure 2:**
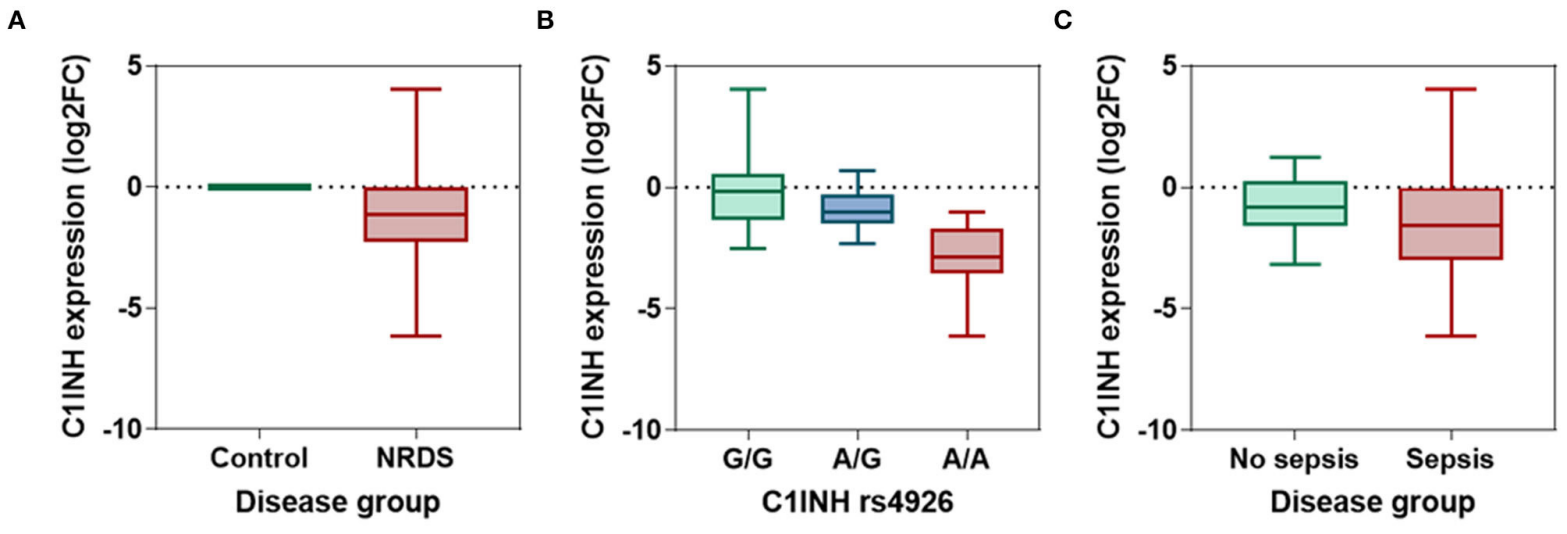
The relative expression level of circulatory *C1INH* gene. **(A)** The log fold change of *C1INH* gene in neonatal lung disease patients (*n* = 94) compared to matched controls (*n* = 45). The expression level was estimated using the LIVAK method and log-transformed. Mann-Whitney *U*-test was used. *p*-value was <0.001. **(B)** Comparison between the expression level of *C1INH* in neonatal lung disease patients with G/G (*n* = 47), A/G (*n* = 18), and A/A (*n* = 29) genotypes of rs4926 missense mutation (c.1438G > A; V480M). Kruskal-Wallis test was used. *p*-value was <0.001. **(C)** Comparison between the expression level of *C1INH* in neonatal lung disease patients with (*n* = 52) and without (*n* = 42) sepsis. Mann-Whitney *U*-test was used. *P*-value was 0.0114.

The *C1INH* gene was significantly downregulated in neonatal lung disease patients compared to controls (*p* < 0.001) (Figure 2A). Median fold change was −1.16 (interquartile range: −2.29 to 0.007). The ROC analysis revealed an area under the curve (AUC) of 0.73 (95%CI:0.64-0.82, *p* < 0.001) at the cutoff value of −0.054 (72.3% sensitivity and 92.1% specificity). Discrimination ability was enhanced when combined with circulatory IL-6 and CRP levels (AUC = 0.926, 95%CI = 0.87-0.97).

In neonatal lung disease patients, patients with A/A genotype exhibited the least expression levels (median: −2.86, IQR: −3.55 to −1.71) compared to their counterparts (A/G: median: −1.01, IQR: −1.49 to −0.28; G/G: median: −0.17, IQR: −1.36 to 0.55; *p* < 0.001) (Figure 2B).

### Association of *C1INH* Expression With Clinical Features and Outcomes

Neonatal lung disease patients who developed sepsis in the NICU had significantly lower expression of *C1INH* than those who did not. The median fold change of septic newborns was −1.58 (IQR: −3.0 to −0.025), while those who did not have sepsis had values of median: −0.84 (IQR: −1.59 to 0.26) (*p* = 0.011) (Figure 2C). At the threshold of −1.28, *C1INH* gene expression can differentiate between cohorts with and without subsequent sepsis with 57.7% sensitivity and 64.3% specificity (AUC = 0.652, 95%CI = 0.54-0.76).

**Figure 3:**
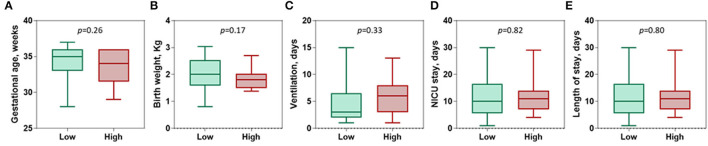
Association of *C1INH* gene expression with disease characteristics. **(A)** Gestational age (weeks). **(B)** Birth weight (Kg). **(C)** Ventilation use (Days). **(D)** Neonatal intensive care unit (NICU) stay (days). **(E)** Length of stay (days). Patients were categorized according to the optimum log fold change of circulatory level of the *C1INH* gene of −0.054 into low expression (*n* = 67) and high levels (*n* = 25). Mann-Whitney *U*-test was used. *P*-value was set significant at < 0.05.

Next, patients were categorized according to the optimum log fold change of circulatory level of the *C1INH* gene into low expression (*N* = 67) and high expression (*N* = 25) sub-groups. There was no observed difference between neonates with different gestational age (median = 35 weeks, IQR = 33-36 vs. median = 34 weeks, IQR = 31.5-36, *p* = 0.26) and birth weight (median = 2.01 Kg, IQR = 1.58-2.53 vs. median = 1.8 Kg, IQR = 1.5-2.03, *p* = 0.17). Additionally, there was no effect on duration of mechanical ventilation (median = 3 days, IQR = 2-6.5 vs. median = 6 days, IQR = 3-8, *p* = 0.33), NICU stay (median = 10 days, IQR = 5-16 vs. median = 11 days, IQR = 7-14, *p* = 0.82), and total hospital days (median = 10 days, IQR = 5-16.5 vs. median = 11 days, IQR = 7-14, *p* = 0.80) (Figure 3).

### Predictor Risk Factors for Sepsis in Neonatal Lung Disease

**Figure 4:**
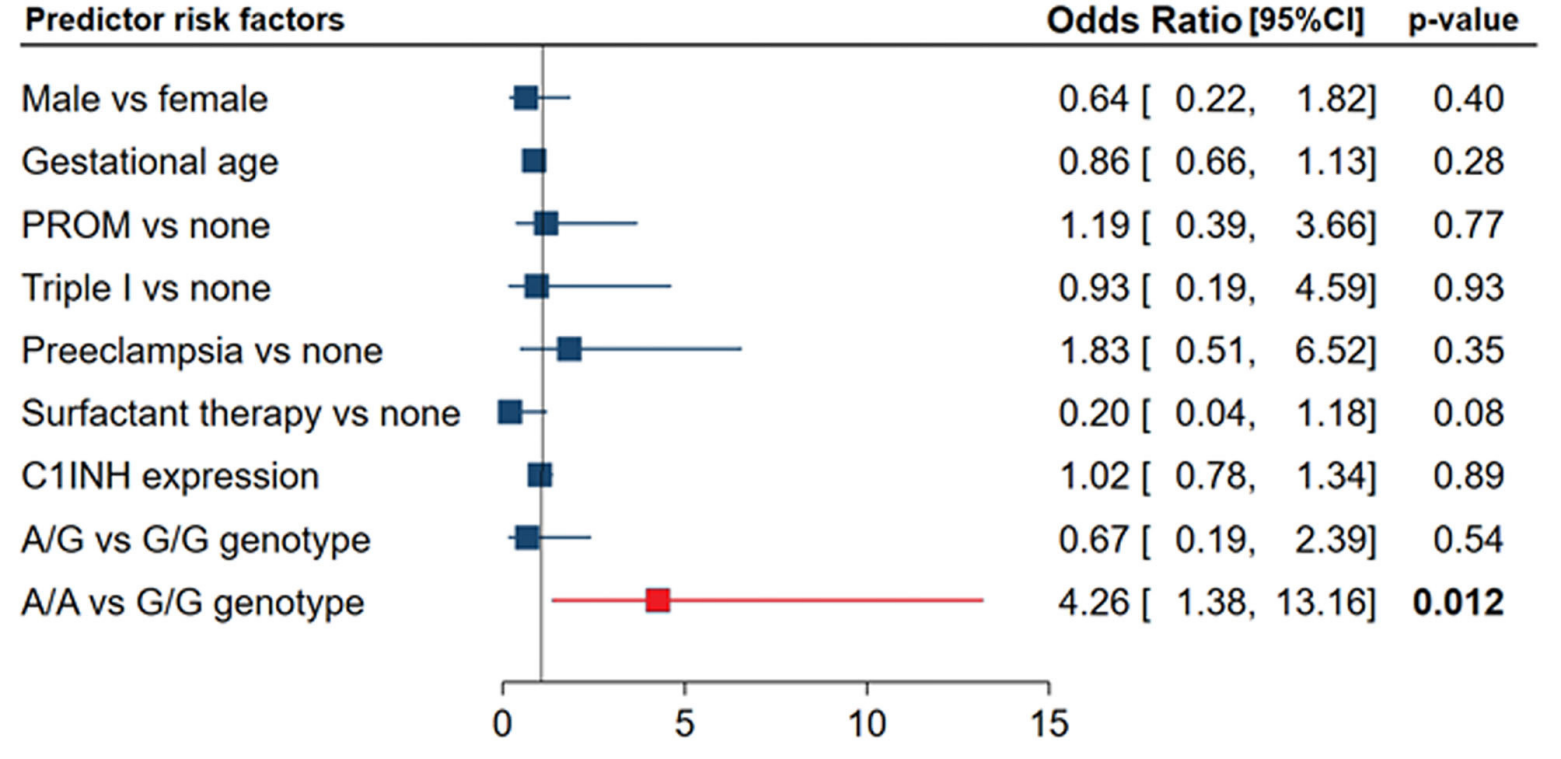
Independent risk factors for developing sepsis in neonatal lung disease patients. Logistic regression analysis was applied. Data are reported and plotted as odds ratio and 95% confidence interval (CI). PROM, Premature rupture of membrane. Data showed homogeneity of the mutant allele (A allele) was associated with four times more risk of developing sepsis (*p* = 0.012).

After adjustment, regression analysis revealed rs4926^*^A/A genotype as an independent predictor risk factor for neonatal sepsis in neonatal lung disease patients (OR = 4.26, 95%CI = 1.38-13.1, *p* = 0.012) (Figure 4). In contrast, gender, gestational age, maternal illness, or receiving postnatal surfactant therapy did not confer higher susceptibility for developing sepsis in our neonatal lung disease cohort.

### Predictor Risk Factors for Mortality in Neonatal Lung Disease

**Figure 5:**
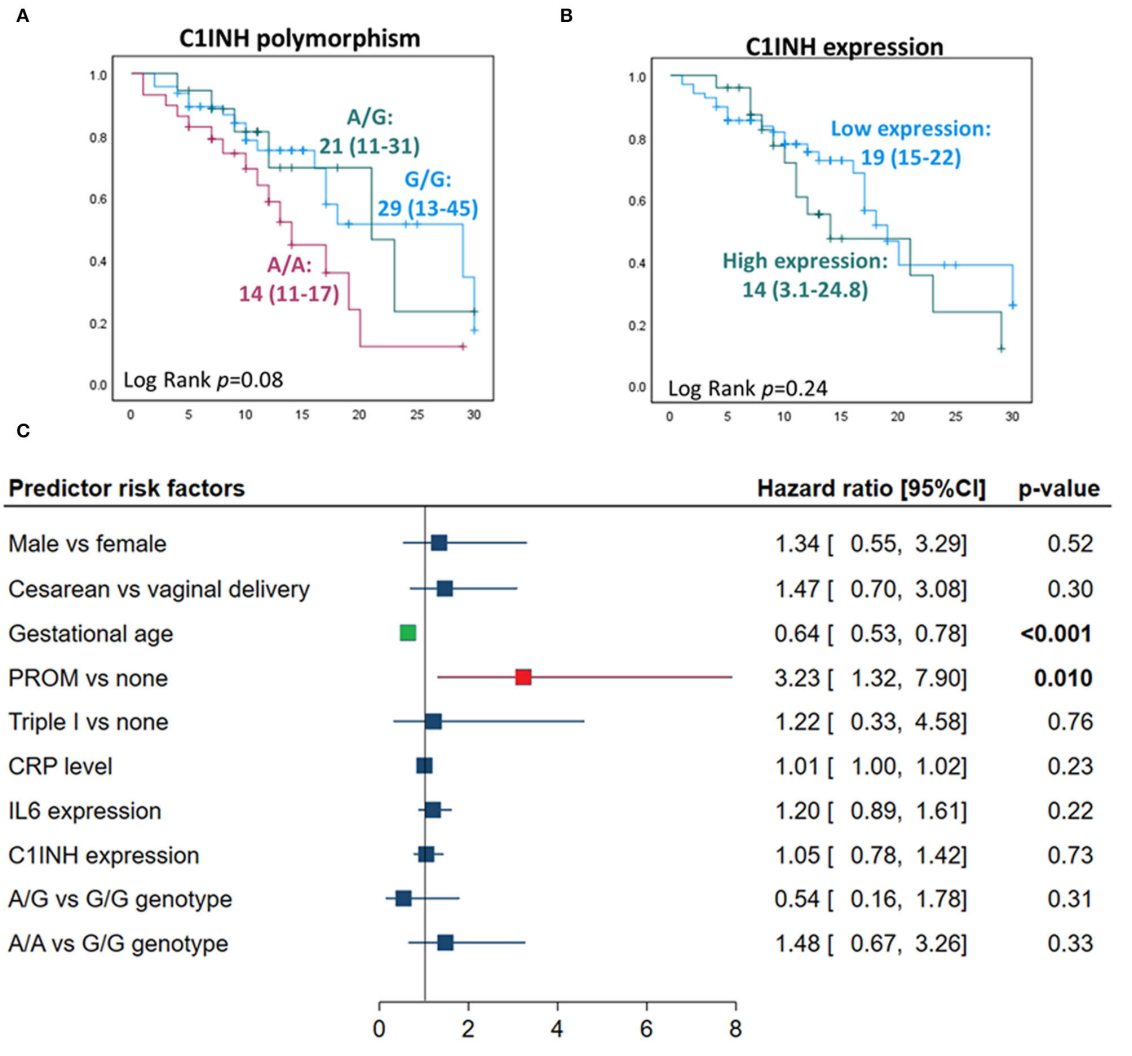
Predictors for mortality in neonatal lung disease patients. **(A)** Kaplan-Meier curve analysis shows *C1INH* gene polymorphism rs4926 (G/A) with neonatal mortality. Log Rank (Mantel-Cox) test was used for comparison. **(B)** Kaplan-Meier curve analysis showing the association of circulatory *C1INH* gene expression with neonatal mortality. Patients were categorized according to the optimum log fold change of circulatory level of the *C1INH* gene of −0.054 into low expression (*n* = 67) and high levels (*n* = 25). **(C)** Cox proportional hazards regression analysis revealed independent risk factors for neonatal mortality in neonatal lung disease patients. Hazard ratio and 95% confidence interval (CI) are reported. Correlation analysis was performed to exclude highly correlated variables from the model (absolute correlation coefficient of >0.5).

There was no significant predictive role of *C1INH* rs4926 polymorphism or circulatory gene expression level between deceased neonates and survivors (Figures 5A,B). Multivariate analysis depicted premature rupture of membrane (HR = 3.23, 95%CI = 1.32-7.9, *p* = 0.010) and low gestational age (HR = 1.56, 95%CI = 1.28-1.88, *p* < 0.001) to be associated with a higher risk of mortality in neonatal lung disease patients (Figure 5C).

## Discussion

Despite the advances in supportive care, neonatal lung disease is still one of the devastating neonatal disorders that results from severe inflammation in the lungs leading to respiratory failure (29). Immune system function, specifically complement function, has been studied regarding prematurity and sepsis (30, 31). Accumulating evidence in the literature has shown that the risk of respiratory distress and genetic background are closely related, and the “gene-host” and “gene-environment” interactions are implicated in the morbidity/mortality associated with this disorder (17, 32–34). In this study, we identified for the first time, up to the authors' knowledge, the *C1INH* rs4926G>A (Val480Met) variant implication in neonatal lung disease risk and outcome in the population under the study. The A/A genotype carriers were two times more likely to develop neonatal lung disease under homozygote and recessive genetic models. Also, a higher frequency of the rs4926^*^A/A genotype was associated with sepsis development in the neonatal lung disease cohort and considered an independent predictor risk factor for neonatal sepsis by regression analysis.

Although this variant is classified as one of the *C1INH* benign variants (https://www.ncbi.nlm.nih.gov/clinvar/variation/254786/) and yielded conflicting results with age-related macular degeneration (35), interestingly, it showed a trend of association with susceptibility to staphylococcal aureus colonization of the nose (36). The latter researchers found an increased prevalence of the G/G genotype in noncarriers than persistent carriers.

Despite previously no differences between the carriers of wild (Val 480) and the mutant (Met 480) C1INH protein were observed concerning “structure, protein stability, serum concentration or inhibition of C1INH protein” (37), however, in the current study, we found a significant association of the studied variant with circulating mRNA transcript levels. In the neonatal lung disease cohort, neonates with the A/A genotype exhibited the least expression levels of *C1INH* mRNA compared to their counterparts (G/G and G/A). This interesting finding is congruent with that the rs4926 variant is identified as one of the cis-expression quantitative trait locus (cis-eQTL) for the *C1INH* gene (https://www.eqtlgen.org/cis-eqtls.html) (38). This indicates that the specified variant could partly explain the RNA expression variation across conditions (39).

C1INH is a highly glycosylated plasma serine-protease inhibitor (105 KDa) and is considered an acute-phase protein that targets several physiological pathways, including the complement activation, blood coagulation (factor XIIa), platelet degranulation, fibrinolysis, and the contact system protease kallikrein (https://www.uniprot.org/uniprot/P05155). Recently, CIINH has been implicated in neutralizing the extracellular histones (40), which are considered as significant components of “damage-associated molecular patterns,” released after cell death, in particular, from the inflammatory cells such as neutrophils during the “neutrophil extracellular trap; NET” process (41). Previous reports confirmed the inflammatory role that the released extracellular histones play in several disorders, including sepsis, acute lung injury, and acute respiratory distress syndrome (40, 42, 43).

**Figure 6:**
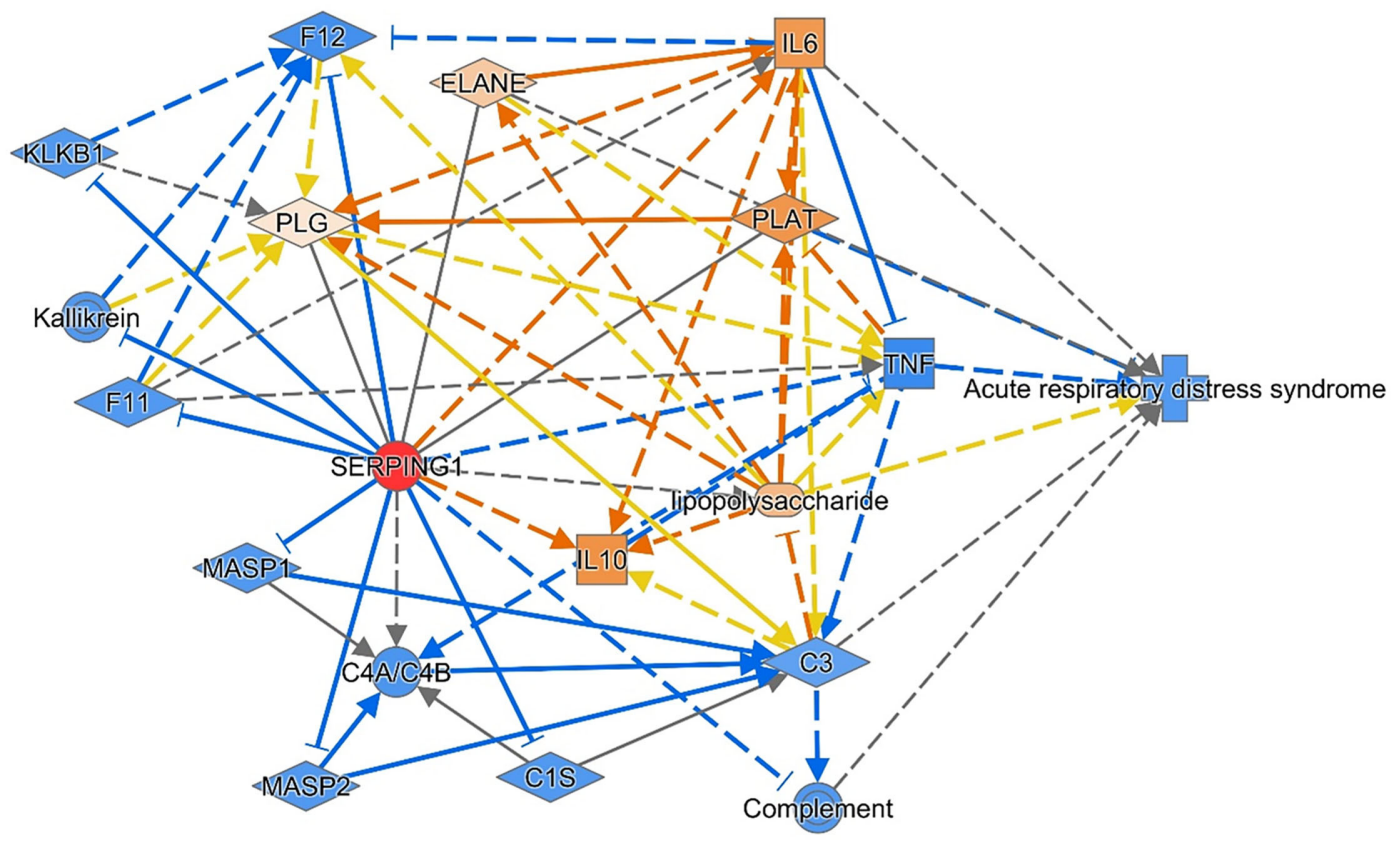
The consequence of the administration of C1INH therapeutic agents is predicted to ameliorate respiratory distress. Reversing the transcriptomic signature of the *C1INH* (*SERPING1*) gene was predicted to ameliorate the disease through multiple mechanisms and pathways. The data are retrieved from knowledge-based text mining using Ingenuity Pathway Analysis (IPA) software (Qiagen Co., USA). Red node: the manipulated gene; orange nodes: predicted to be upregulated; and blue nodes: predicted to be downregulated. IL, Interleukin; C, complement; F, clotting factor; KLKB1, Kallikrein B1; MASP1, mannose-associated serine protease 1; PLAT, Plasminogen Activator, Tissue Type; PLG, Plasminogen; TNF, tumor necrosis factor.

Dependent on the C1INH carbohydrate structure (associated with an excessive negative charge), the protease inhibition capability, endotoxin scavenging property, and leukocyte rolling inhibition, C1INH could be a promising protective biomolecule that prevents the deleterious effects of exaggerated inflammation in acute respiratory distress (40). As illustrated in Figure 6, the administration of C1INH therapeutic agents is predicted to ameliorate the respiratory distress *via* several molecular pathways, such as inhibition of the classical complement enzymes (CIs), the activated factors XIIa/XIa, plasma kallikrein, plasmin, tissue-type plasminogen activator (tPA) and thrombin (44). C1INH inhibits the lectin pathway of complement activation *via* inactivation of “mannan-binding lectin-associated serine protease-1 and−2 (MASP1 and MASP2)” and inhibits the alternative pathway of activation by binding to C3b. In this sense, C1-INH is a significant regulator of all three pathways of complement activation (45). Given the heavily glycosylated structure of C1INH and bearing a “sialyl Lewis-related moiety” through which it binds to endothelial cell-surface selectins E and P, in competition with white blood cells binding, it can exert its anti-inflammatory function (46). These data could support the current study observations of decreased C1INH circulatory transcript levels in neonatal lung disease cases (with a subsequent decline in its protective roles) compared to healthy neonates and could explain, in part, the association of the risky allele genotype (AA) carriers with less circulatory gene transcript expression levels and increased susceptibility to be complicated by sepsis. Due to limited blood sampling of the enrolled neonates, the C1INH protein levels assessment was not feasible to correlate with the circulating transcript expression. Hence, further replication studies that quantify both C1INH protein and transcript levels are required to confirm the study findings.

The ROC analysis revealed a good area under the curve for circulatory C1INH to discriminate neonatal lung disease group from healthy neonates, and this discriminative ability was enhanced when combined with the traditional circulatory marker levels of IL-6 and CRP. This finding confirms that no single biomarker can predict outcome in acute respiratory distress patients, as several different pathways are involved in disease development (47). The acute phase reactant CRP was extensively studied in several contexts, including neonatal lung disease and neonatal sepsis, and it remains the preferred biomarker in many NICUs (48, 49). Also, the pleiotropic pro-inflammatory cytokine IL-6 exhibited a prolonged response to the pathogenic challenge, on several occasions, compared to other cytokines (50, 51). It was implicated in multiple biological processes, including T cell maturation/differentiation, cell survival, apoptosis, and inflammation (52). Taken together, we can conclude that C1INH exp could be an ancillary test with the currently used biomarkers like IL6 and CRP based on our results.

It is worth noting that the authors referred to some studies related to acute respiratory distress cases in the discussion section (as there was a limitation of available literature that explore the association of the studied gene with neonatal lung disease) to point to the potential similarity of the inflammatory process and cascade seen in neonatal lung disease vs. the former cases, considering the different etiopathology and age of presentation between both disorders.

Despite the preventive and therapeutic measurements followed in our NICU, there was a high rate of late-onset sepsis in our cohort. It is a challenge that the pediatricians face in our local NICU and can be attributed to several factors, including low resources affecting the availability of personal protective equipment, more strains of antibiotic-resistance bacteria, and lack of Group B Strep (GBS) screening of mothers. This concern has been raised by Amer et al. as “one of the unfinished agenda in developing countries” (53). It is also of importance to emphasize the Molloy et al. notion in that “the lack of the majority of clinical signs/symptoms complicate the identification of sepsis on the neonate. The difference in the NICU is that it may be difficult to differentiate contamination from true infection, and the long-term impact of these infections is greater on the developing brain. Therefore, a consensus definition is required that can be universally generalizable and validated in international datasets and correlated with neurodevelopmental outcomes” (54).

The main limitations of this study are the relatively small sample sizes, the lack of simultaneous investigation of other possible SNPs that could influence the neonatal lung disease risk and outcome, and the characteristics of the control group. Furthermore, due to the small volume of blood was available in this work, the authors could only investigate the *SERPING1* transcript expression. It would be helpful to demonstrate also the SERPING1 protein level in future studies as “the mRNA level may not always reflect the corresponding protein expression due to the posttranscriptional processing” (45). However, our findings highlight the potential role of the *C1INH* rs4926 variant in increased neonatal lung disease risk and prediction of sepsis development in this type of preterm infants cohort. Also, this variant could impact gene expression, which supports its role in neonatal lung disease etiopathology. Nevertheless, additional more extensive genomic and functional studies are required to confirm the study conclusions.

## Data Availability Statement

The original contributions presented in the study are included in the article/Supplementary Material, further inquiries can be directed to the corresponding author/s.

## Ethics Statement

The studies involving human participants were reviewed and approved by the Ethics Committee of Suez Canal University, Faculty of Medicine, Ismailia, Egypt (Approval No. 3435). Written informed consent to participate in this study was provided by the participants' legal guardian/next of kin.

## Author Contributions

All authors listed have made a substantial, direct, and intellectual contribution to the work and approved it for publication.

## Conflict of Interest

The authors declare that the research was conducted in the absence of any commercial or financial relationships that could be construed as a potential conflict of interest.

## Publisher's Note

All claims expressed in this article are solely those of the authors and do not necessarily represent those of their affiliated organizations, or those of the publisher, the editors and the reviewers. Any product that may be evaluated in this article, or claim that may be made by its manufacturer, is not guaranteed or endorsed by the publisher.
