# Supplementary material for: Component 1 Inhibitor Missense (Val480Met) Variant Is Associated With Gene Expression and Sepsis Development in Neonatal Lung Disease
Source: Front Pediatr. 2022 May 20;10:779511. doi: 10.3389/fped.2022.779511 (PMC9163386; doi:10.3389/fped.2022.779511)
Supplement: Supplementary file 1 [file Image_1.pdf]

Supplementary Material

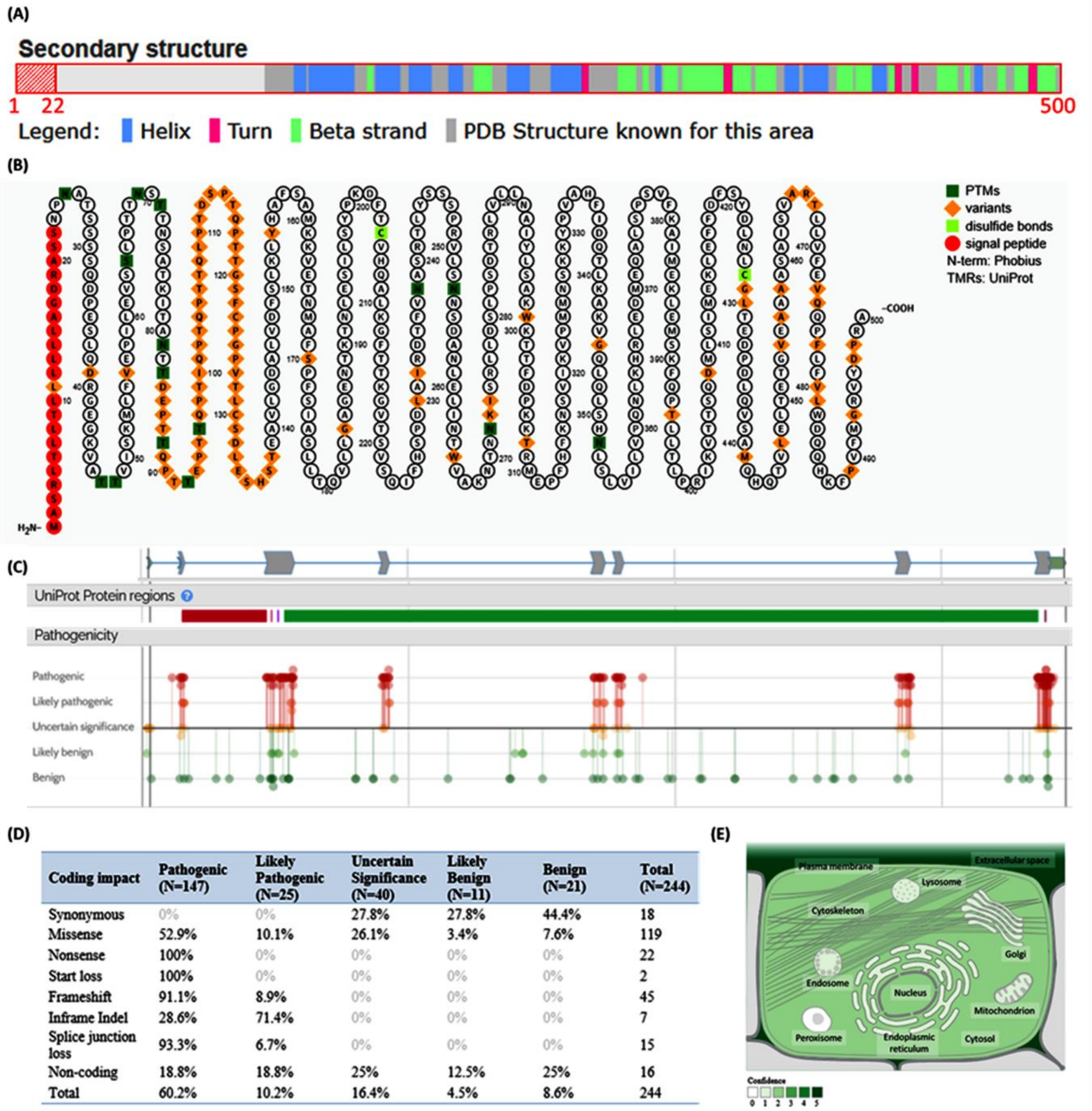

Coding impact	Pathogenic (N=147)	Likely Pathogenic (N=25)	Uncertain Significance (N=40)	Likely Benign (N=11)	Benign (N=21)	Total (N=244)
Synonymous	0%	0%	27.8%	27.8%	44.4%	18
Missense	52.9%	10.1%	26.1%	3.4%	7.6%	119
Nonsense	100%	0%	0%	0%	0%	22
Start loss	100%	0%	0%	0%	0%	2
Frameshift	91.1%	8.9%	0%	0%	0%	45
Inframe Indel	28.6%	71.4%	0%	0%	0%	7
Splice junction loss	93.3%	6.7%	0%	0%	0%	15
Non-coding	18.8%	18.8%	25%	12.5%	25%	16
Total	60.2%	10.2%	16.4%	4.5%	8.6%	244

Supplementary Figure S1. Protein structure and the genetic variants of the *C1INH gene*. (A) Secondary structure of C1INH protein showing signal peptide (1-22 residues) and protein chain (23-500 residues). Unlike most Serpin family members, C1-inhibitor has a 2-domain structure. The C-terminal serpin domain is similar to other serpins, which is the part of the C1-inhibitor that provides the inhibitory activity. The N-terminal tail is not essential for C1-inhibitor to inhibit proteases. This domain has no similarity to other proteins. [Data source: UniProt.org]. (B) Annotated sequence feature of C1INH protein. C1-inhibitor is highly glycosylated, bearing both N- and O-glycans. N-terminal domain is especially heavily glycosylated. Signal peptide residues are shown in red. Change amino

acid from Valine (V) to Methionine (M) at position 480 (V480M, p.Val480Met). [Data source: Protter; http://wlab.ethz.ch/protter/]. (C-D) Variant distribution across the gene and their pathogenicity. (D) Percentage of different types of polymorphisms. (E) Subcellular localization of C1INH protein. [Data source: Compartments Subcellular Localization database; https://compartments.jensenlab.org].
